# Prevalence of CCR7‐Positive CD8 T Cells as a Prognostic Factor in B‐Cell Maturation Antigen ‐Targeted Chimeric Antigen Receptor T Cell Therapy

**DOI:** 10.1002/jha2.70040

**Published:** 2025-05-05

**Authors:** Yoshiaki Marumo, Masaki Ri, Toru Ebina, Tomoyuki Nakamura, Yoshiko Oshima, Takahiro Nakashima, Shiori Kinoshita, Tomotaka Suzuki, Tomoko Narita, Takaomi Sanda, Hirokazu Komatsu, Shinsuke Iida

**Affiliations:** ^1^ Department of Hematology and Oncology Graduate School of Medical Sciences Nagoya City University Nagoya Aichi Japan

**Keywords:** BCMA‐targeting, CAR‐T cells, myeloma, T cell stemness, corresponding author: Masaki RI: rrmasaki.sub@gmail.com

## Abstract

**Background:**

Chimeric antigen receptor T (CAR‐T) cell therapy is effective for relapsed or refractory multiple myeloma (RRMM); however, relapse after the B‐cell maturation antigen (BCMA) CAR‐T cell therapy is associated with poor outcome. Hence, appropriate biomarkers that can predict the outcome are needed.

**Methods:**

Patients who received idecabtagene vicleucel, a BCMA‐targeted CAR‐T cell therapy, were divided into two groups according to a cut‐off value of 180 days for the progression‐free survival (PFS) event.

**Results:**

Patients in the short responder group were older at diagnosis, had a shorter time from diagnosis to apheresis, and more frequently had prior bispecific antibody treatment or alkylator‐containing chemotherapies, while they received less immunomodulatory drugs‐based chemotherapy just prior to apheresis. Apheresis samples collected from the long responder group had significantly higher proportion of CD8‐positive naïve or stem cell memory (CCR7^+^CD45RO^−^) or central memory (CCR7^+^CD45RO^+^) T cells. When these two T cell subsets were combined into CCR7‐positive CD8 T cells, the patients with high levels of CCR7‐positive CD8 T cells showed significantly better PFS.

**Conclusion:**

In the future, our results will help us to select specific patients that are likely to have a more favorable outcome and should contribute to establishing an optimal application strategy for CAR‐T cell therapies in RRMM.

## 1 Introduction

Multiple myeloma (MM) is an incurable plasma cell neoplasm [1, 2]. Although advancements in treatment, such as proteasome inhibitors (PIs), immunomodulatory drugs (IMiDs), anti‐CD38 monoclonal antibodies, have led to prolonged survival, patients who are refractory to these drugs, in particular triple‐class refractory cases, have poor outcomes (median progression‐free survival [PFS] of 6.2 months) with subsequent regimens [3].

B‐cell maturation antigen (BCMA) is a member of the tumor necrosis factor receptor superfamily that has a key role in the proliferation, maturation, and differentiation of B cells. BCMA is nearly universally expressed on myeloma cells [4], and thus is a promising target antigen for the treatment of MM. Idecabtagene vicleucel (ide‐cel) is one of the BCMA‐directed chimeric antigen receptor T (CAR‐T) cell therapy drugs showing durable responses in triple class exposed, heavily treated patients with relapsed or refractory multiple myeloma (RRMM), and it has been approved for use in the United States [5], Europe, and Japan. However, disease progression after the BCMA‐CAR T cell therapy remains a significant clinical problem, because of the consequent worse outcome (median PFS for the first salvage therapy after CAR‐T relapse was 3.5 months) [6]. Several factors have been identified in clinical trials that might influence the response to CAR‐T therapy, such as the presence of extramedullary disease (EMD) or high‐risk cytogenic abnormalities, and poor performance status (PS) at lymphodepleting chemotherapy (LDC) [7]. Previous reports have revealed a high risk of early relapse in patients stratified into three independent risk groups (MyCARe model) [8]. However, prediction of the efficacy of the ide‐cel therapy remains challenging due to the diversity of patient characteristics and differences in disease or immunological status, we need more appropriate biomarkers associated with outcome.

The quality of apheresed T cells is considered the most important of the factors determining the quality of CAR‐T cells [9], and early lineage phenotypes are particularly essential for maintaining T cell fitness. However, cumulative dose of chemotherapy could reduce the apheresis product (AP) quality [10]. A previous report showed that the median percentage of CD45RO^−^CD27^+^ CD8 T cells was lower in peripheral blood of patients with RRMM than those with RRMM post‐induction [11]. In previous reports assessing the association of the T cell AP with clinical response to CD19 CAR‐T cell therapy [12], early responders to CD19 CAR‐T cell therapy had a higher proportion of CD8 naïve or stem cell memory T cells than non‐responders when analyzed by flow cytometry or single‐cell RNA sequencing [9, 13]. In terms of BCMA CAR‐T cell therapies, post hoc analysis of phase I ide‐cel trial data showed that the population of naïve CD4 cells was related to longer PFS [14]; however, no real‐world data on this population are currently available.

Here, we assessed whether the T cell composition of apheresis samples from patients treated with ide‐cel at our institute and clinical baseline characteristics of the treated patients can affect or predict the length of PFS after the ide‐cel therapy.

## 2 Method

### 2.1 Patient and Sample Collection

This was a retrospective single‐center observational study of patients who received ide‐cel for RRMM after treatment with PIs, IMiDs, and anti‐CD38 antibodies. The treatment protocol including LDC, a combination of cyclophosphamide with fludarabine, was in accordance with the KarMMa study [5]. Data and sample collection were approved by the institutional ethics committee (protocol #70‐24‐0005). All patients were given the opportunity to refuse participation. The samples at leukapheresis were collected and cryopreserved after red blood cell hemolysis as APs.

### 2.2 Clinical Data Assessment

Clinical data were extracted from medical records. High‐risk cytogenetics were defined by the presence of del(17p), t(4;14), t(14;16), and 1q gain or amplification [15]. Plasma cell leukemia (PCL) was defined in accordance with the updated consensus statement [16] and EMD was defined as organ manifestation assessed with computed tomography (CT) scan, magnetic resonance imaging, or positron emission tomography/CT, and sole paraskeletal involvement was excluded from that definition. Patient responses were monitored in outpatient clinics and evaluated based on the International Myeloma Working Group (IMWG) criteria [17]. Patients were divided into two groups according to the number of days until the PFS event occurred (a long responder group [PFS≧180 days] and short responder group [PFS<180 days]) as previously reported [18]. The concentration of soluble BCMA (sBCMA) was analyzed in the plasma samples of each patient on initiation of LDC using a Human BCMA DuoSet ELISA Kit (DY193, R&D Systems, Waltham, MA, USA), with the mean value of triplicate samples calculated for each specimen.

### 2.3 Flow Cytometric Analysis

The cryopreserved APs were thawed and incubated with Human Fc Block reagent (from Becton Dickinson, BD, Heidelberg, Germany). For the analysis, cells were washed with PBS and stained using a Zombie Red Fixable Viability Kit (Biolegend, San Diego, CA, USA) for 10 min at room temperature, and then washed with Stain Buffer supplemented with Fetal Bovine Serum (BD). They were subsequently incubated with the following antibodies: PerCP/Cy5.5‐CD3, BV510‐CD4, APC/H7‐CD8, BV650‐CCR7, PE/Cy7‐D45RO, and BV786‐PD‐1 (all from Biolegend) in 100 µL of antibody dilution buffer for 15 min at 4°C. After washing, cells were analyzed using an FACSymphonyA1 flow cytometer (BD). Between 0.5 and 1 million CD3‐positive live cell singlet events were acquired per sample. Data analysis was performed using Kaluza software (Beckman Coulter, Brea, CA, USA). For adequate gating, we used fluorescence minus one control and the gating strategy is shown separately (Figure S1).

### 2.4 Statistical Analysis

PFS was defined as the time from CAR T‐cell infusion to disease progression or death. Patients who did not experience such events were censored at the date of the final follow‐up. Kaplan–Meier curves for PFS were generated, and the log‐rank test was used to compare differences between subgroups. Univariable Cox regression analyses were performed to identify the risk factors associated with PFS. The Mann–Whitney *U*‐test was used to compare continuous variables, and the Fisher exact test was used to analyze categorical variables. The threshold for significance was set at *p* < 0.05. All statistical analysis was performed using EZR (version 1.55) in R commander [19].

## 3 Results

### 3.1 Patient Characteristics

**FIGURE 1:**
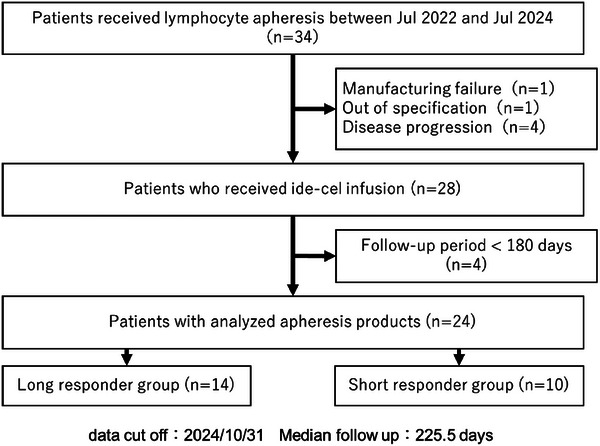
Patient flowchart. A total of 34 patients were enrolled and underwent leukapheresis from July 2022 to April 2024 at our institute. Six patients were excluded from the analysis due to disease progression before ide‐cel infusion (*n* = 4), manufacturing failure (*n* = 1), and leukapheresis material out of specification (*n* = 1). Twenty‐eight patients were successfully administered ide‐cel. The last patient had the ide‐cel infusion on July 8, 2024. We analyzed 24 patients who were followed for at least 180 days after administration of ide‐cel, and 14 and 10 patients were assigned to the long responder group and short responder group according to length of progression‐free survival, respectively.

A total of 34 patients were enrolled and underwent leukapheresis from July 2022 to April 2024 at our institute. Six patients were excluded from the analysis due to disease progression before ide‐cel infusion (*n* = 4), manufacturing failure (*n* = 1), or out of specification drug product (*n* = 1). Twenty‐eight patients successfully received ide‐cel. The last patient had the ide‐cel infusion on July 8, 2024. We analyzed 24 patients who were followed for at least 180 days after administration of ide‐cel. The data cut‐off was October 31, 2024, the median follow‐up having been 225.5 days from CAR‐T cell infusion. At the data cut‐off point, 10 patients had an on‐going response; and 14 and 10 patients were assigned to the long responder and short responder group according to the time of PFS, respectively (Figure 1). Our cohort included no patients with PCL.

Table 1 shows the baseline characteristics of patients in the overall study population and in the groups. The age at diagnosis and at apheresis of patients in the overall population was 60 and 65 years old, respectively, and the long responder group included patients with significantly younger age at diagnosis (57 years old vs. 63.5 years old, *p* = 0.030) and longer time from diagnosis to apheresis (86 months vs. 54 months, *p* = 0.019). In terms of treatment history, the short responder group contained a greater proportion of patients who had prior bispecific antibody treatment (40% vs. 0%, *p* = 0.020) or alkylator‐containing chemotherapy just before apheresis (40% vs. 0%, *p* = 0.020), but a smaller proportion of patients who received IMiDs‐based chemotherapy just before apheresis (10% vs. 64%, *p* = 0.013).

**TABLE 1:** Baseline characteristics of all study participants and long or short responder groups.

	All participants (*n* = 24)	Long responders (*n* = 14)	Short responders (*n* = 10)	*p*‐Value
Age at apheresis (median, range)	64 (46–77)	63 (46–77)	68 (51–75)	0.121
Age at diagnosis (median, range)	59.5 (31–72)	55 (31–72)	63 (49–72)	0.037
Gender (male, %)	12 (42.3%)	8 (44.4%)	4 (40.0%)	1.000
Median time from diagnosis to apheresis (months, range)	63.5 (9–292)	84 (15–292)	47 (9–135)	0.019
Poor‐risk cytogenetic abnormality (*n*, %)	22 (78.6%)	12 (66.7%)	10 (100.0%)	0.202
ECOG‐PS≧2 at LDC (*n*, %)	11 (39.3%)	5 (27.8%)	6 (60.0%)	0.238
Presence of extramedullary disease (*n*, %)	13 (46.2%)	9 (50.0%)	4 (40.0%)	0.708
High tumor burden at LDC (*n*, %)	2 (7.1%)	2 (11.1%)	0 (0.0%)	0.524
Median soluble BCMA at LDC (ng/mL, range)	203.3 (7.20–6993)	144.1 (25.1–5981)	274.1 (7.20–6993)	1.000
Elevated ferritin level at LDC (*n*, %)	14 (58.3%)	7 (53.8%)	7 (87.5%)	0.174
Infused CAR^+^T cells (×10^6^ cells, median, range)	449.25 (346.16–532.94)	448.8 (368.45–532.94)	449.65 (346.16–522.40)	0.715
Number of prior regimens (median, range)	5 (3–13)	5 (3–13)	5 (4–10)	0.929
Prior ASCT (*n*, %)	26 (92.9%)	18 (100.0%)	8 (80.0%)	0.126
Triple‐class refractory (*n*, %)	26 (92.9%)	17 (94.4%)	9 (90.0%)	1.000
Penta‐refractory (*n*, %)	9 (32.1%)	6 (33.3%)	3 (30.0%)	1.000
Prior bispecific antibody (*n*, %)	4 (14.3%)	0 (0.0%)	4 (40.0%)	0.010
BCMA‐targeting	2 (7.1%)	0 (0.0%)	2 (20.0%)	
GPRC5D‐targeting	2 (7.1%)	0 (0.0%)	2 (20.0%)	
Treatment‐free interval over 3 months (*n*, %)	15 (53.6%)	12 (66.7%)	3 (30.0%)	0.114
Systemic treatment before apheresis				
PIs‐based	10 (35.7%)	6 (33.3%)	4 (40.0%)	1.000
IMiDs‐based	13 (46.4%)	12 (66.7%)	1 (10.0%)	0.006
Alkylator‐containing	4 (14.3%)	0 (0.0%)	4 (40.0%)	0.010

Abbreviations: ECOG‐PS, Eastern Cooperative Oncology Group performance status; ASCT, autologous stem cell therapy; BCMA, B cell maturation antigen; CAR^+^T, chimeric antigen receptor positive T; GPRC5D, G protein‐coupled receptor class C group 5 member D; IMiDs, immunomodulatory drugs; LDC, lymphodepleting chemotherapy; PIs, proteasome inhibitors.

### 3.2 Cell Composition of Apheresis Samples

In order to investigate the relationship of the cell composition of APs with clinical factors between the groups, we assessed the four CD4 or CD8 T cell subsets in APs (naïve or stem cell memory [CCR7^+^CD45RO^−^]; central memory [CCR7^+^CD45RO^+^]; effector memory [CCR7^−^CD45RO^+^]; effector [CCR7^−^CD45RO^−^]) using flowcytometric analysis, and then PD‐1 positive subsets.

**FIGURE 2:**
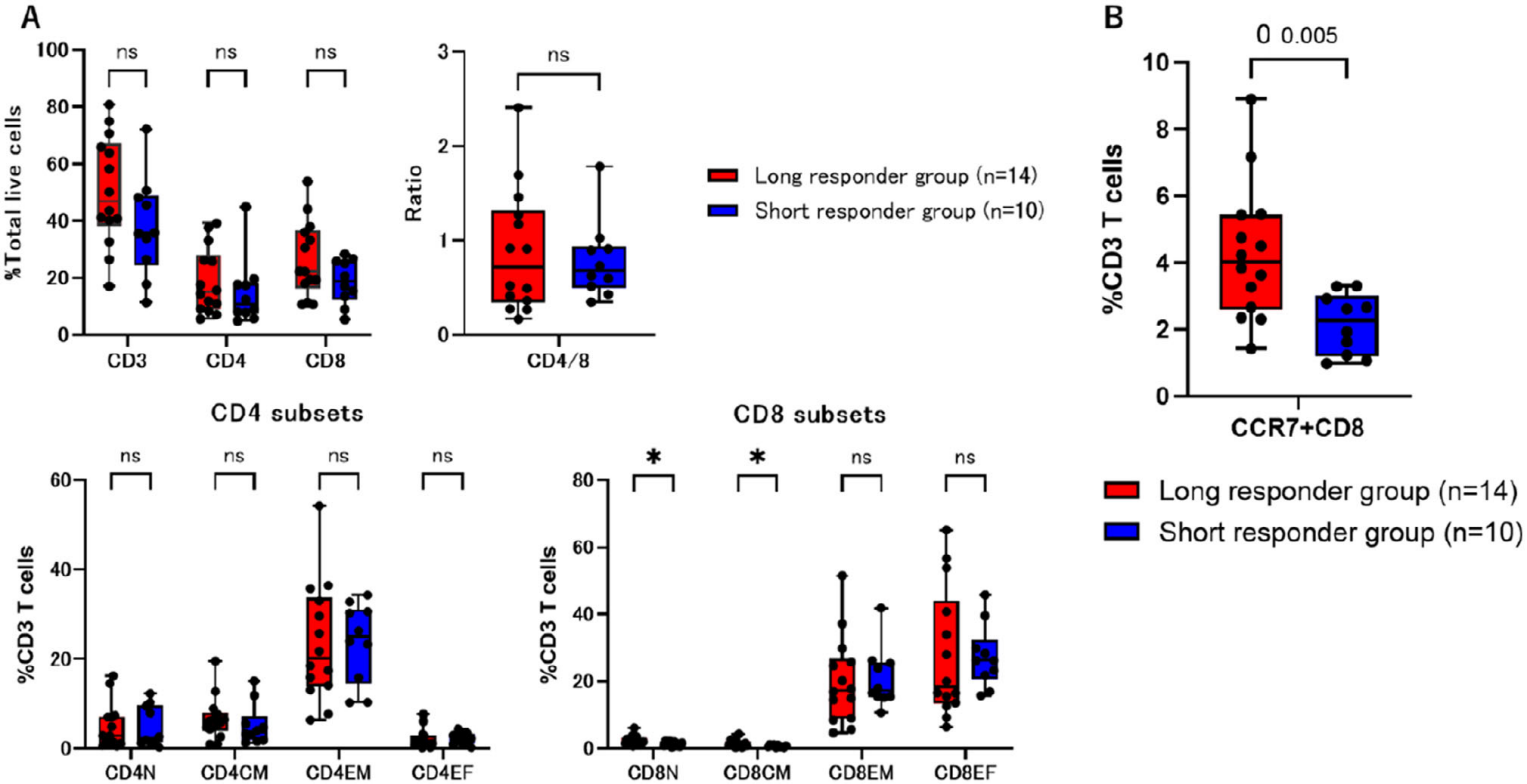
T‐cell composition of apheresis samples in the long or short responder group Flow cytometric analysis of apheresis samples was performed. (A) While the frequency of CD3, CD4, or CD8 and the CD4:CD8 ratio were not different, significantly more cells in the CD8 naïve or stem cell memory subset (CCR7^+^CD45RO^−^, 2.49% vs. 1.51%, *p* = 0.026) and central memory subsets (CCR7^+^CD45RO^+^, 1.30% vs. 0.71%, *p* = 0.019) were detected in the long responder group, with no difference in CD4 cell subsets. (B) As the frequencies of cells in the naïve or stem cell memory and central memory subsets of CD8 T cells were correlated (Spearman's rank correlation coefficient, 0.431, *p* = 0.037), we combined these subsets into CCR7‐positive CD8 T cell subsets. The long responder group had a significantly higher proportion of CCR7‐positive CD8 T cells (4.04% vs. 2.27%, *p* = 0.005). Abbreviations:N, naïve or stem cell memory; CM, central memory; EM, effector memory; EF, effector.

Whereas the frequency of CD3, CD4, or CD8 cells and the CD4‐to‐CD8 ratio were not different between groups, significantly larger proportion of CD8 naïve or stem cell memory cells (CD8^+^CCR7^+^CD45RO^−^, 2.49% vs. 1.51%, *p* = 0.026), and CD8 central memory cells (CD8^+^CCR7^+^CD45RO^+^, 1.30% vs. 0.71%, *p* = 0.019) were observed in the long responder group (Figure 2A). The frequency of naïve or stem cell memory and central memory CD8 cells were positively correlated (Spearman's rank correlation coefficient, 0.431, *p* = 0.037). Hence, we then combined these subsets into one CCR7‐positive CD8 T cell subset. Notably, the long responder group, consequently, had a larger proportion of CCR7‐positive CD8 T cells (4.04% vs. 2.27%, *p* = 0.005, Figure 2B). There were no significant differences in levels of CD4 subsets between the two groups.

**FIGURE 3:**
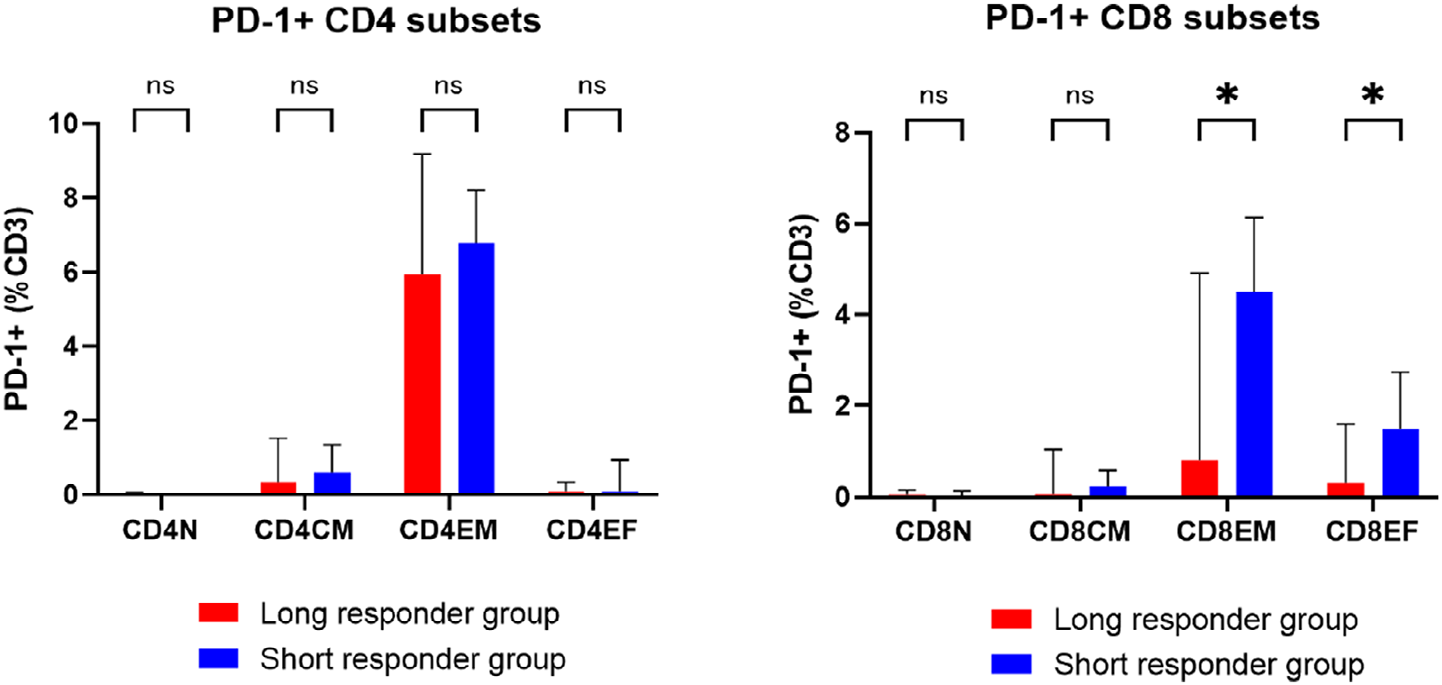
PD‐1 positivity of CD4 or CD8 subsets in the long or short responder group. We assessed PD‐1 positivity of cells in the CD4 or CD8 T cell subsets by flow cytometry. Patients in the long responder group had significantly lower proportion of PD‐1‐positive CD8 effector memory cells (CCR7^−^CD45RO^+^, 0.90% vs. 4.64%, *p* = 0.032) or CD8 effector cells (CCR7^−^CD45RO^−^, 0.35% vs. 1.70%, *p* = 0.046) than the short responder group, while PD‐1‐positive cells did not differ in CD4 cell subsets. Abbreviations:N, naïve or stem cell memory; CM, central memory; EM, effector memory; EF, effector.

We also assessed the PD‐1 positivity of CD4 or CD8 T cell subsets (Figure 3). Although the proportions of PD‐1‐positive CD8 effector memory cells (CCR7^−^CD45RO^+^, 0.90% vs. 4.64%, *p* = 0.032) and CD8 effector cells (CCR7^−^CD45RO^−^, 0.35% vs. 1.70%, *p* = 0.046) were significantly lower in the long responder group, no such between‐group differences in the proportions of PD‐1‐positive CD4 cells were noted.

### 3.3 Efficacy

**FIGURE 4:**
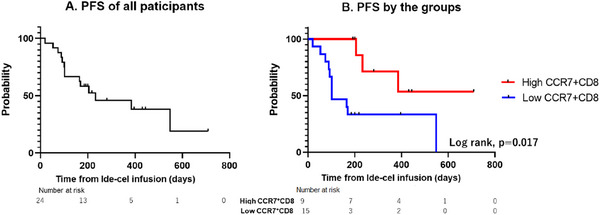
Progression‐free survival of all participants or participants with high‐ or low‐level CCR7^+^CD8 cells. The Kaplan–Meir curve for progression‐free survival of all participants (A) and participants with high‐ or low‐level CCR7+CD8 cells (B).

The overall response rate and CR rate of all participants were 87.5% and 50.0%, respectively, and patients in the long responder group showed significantly higher CR rate than those in the short responder group (78.6% vs. 10.0%, *p* = 0.003, Table S1). The median PFS of all participants was 196 days (Figure 4A). To assess the impact of CCR7‐positive T cells on PFS, we used receiver operating characteristic (ROC) analysis to divide patients into two risk groups (high CCR7^+^CD8 group, *n* = 9, and low CCR7^+^CD8 group, *n* = 15) based on two factors, the proportion of CCR7‐positive CD8 T cells at leukapheresis and the PFS at 180 days from administration of ide‐cel. According to this ROC curve, the cut‐off value was 3.314% (area under the curve, 0.843, 95% confidence interval [CI], 0.686–1.000). Notably, patients with high levels of CCR7‐positive CD8 T cells had significantly better outcomes than those with low levels of CCR7‐positive CD8 T cells (median PFS, NA vs. 102 months, Log rank test, *p* = 0.017, Figure 4B). The characteristics of patients with high‐ and low‐level CCR7‐positive CD8 T cells are shown in Table 2. There was no significant difference in age at apheresis, the number of prior treatments before apheresis, high‐risk cytogenetic abnormality, baseline ferritin level at LDC, time from diagnosis to apheresis, and frequency of treatment‐free intervals over 3 months, but IMiDs‐based chemotherapy tended to be more common in the high‐level CCR7^+^CD8 group, although the difference was not significant (66.7% vs. 26.9%, *p* = 0.092). A previous report proposed a prediction model of post‐BCMA targeting CAR‐T cell therapy (MyCARe model) [8]. We tried to apply this model for our cohort, however we were unable to find significant differences in each risk arm (Figure S2, Log rank, *p* = 0.3298).

**TABLE 2:** Baseline characteristics by the frequency of CCR7‐positive CD8 T cells.

	High CCR7^+^CD8 (*n* = 9)	Low CCR7^+^CD8 (*n* = 15)	*p*‐Value
Age at apheresis (median, range)	62 (46–77)	68 (51–75)	0.107
Age at diagnosis (median, range)	55 (31–72)	62 (47–72)	0.128
Gender (male, %)	4 (44.4%)	6 (40.0%)	1.000
Median time from diagnosis to apheresis (months, range)	80.3 (24–297)	60 (22–152)	0.387
Poor‐risk cytogenetic abnormality (*n*, %)	5 (62.5%)	11 (73.3%)	0.657
Elevated ferritin level at LDC (*n*, %)	3 (33.3%)	4 (33.3%)	1.000
ECOG‐PS≧2 at LDC (*n*, %)	3 (33.3%)	6 (40.0%)	1.000
Number of prior regimens (median, range)	7 (3–13)	5 (3–10)	0.200
Treatment‐free interval over 3 months (*n*, %)	6 (66.7%)	6 (40.0%)	0.400
PIs‐based chemotherapy before apheresis	4 (44.4%)	6 (40.0%)	1.000
IMiDs‐based chemotherapy before apheresis	6 (66.7%)	4 (26.9%)	0.092
Alkylator‐containing chemotherapy before apheresis	0 (0.0%)	4 (26.7%)	0.259

Abbreviatoins: ECOG‐PS, Eastern Cooperative Oncology Group performance status; LDC, lymphodepleting chemotherapy; PIs, proteasome inhibitors; IMiDs, immunomodulatory drugs.

In terms of the treatment response, patients in the high CCR7‐positive CD8 group showed a higher CR rate than those in the low CCR7‐positive CD8 group, but not significantly, which may be due to the number of patients in the high CCR7‐positive CD8 group (Table S2). No correlation was identified between the frequency of CCR7‐positive CD8 T cells and toxicities of the ide‐cel therapy, such as cytokine release syndrome or immune effector cell‐associated neurotoxicity syndrome.

### 3.4 The Treatment Effect on T Cell Subsets in Apheresis Products

Next, to assess the effect of treatment on the cell composition before leukapheresis, we calculated the frequency of CCR7‐positive CD8 T cells for each treatment history. Notably, using IMiDs‐based chemotherapy just prior to apheresis was related to the higher number of CCR7‐positive CD8 T cells (8.25% vs. 4.83%, *p* = 0.014, Figure S3) compared to others.

Similarly, PD‐1‐positive CD4 or CD8 T cell subsets were analyzed. Systemic chemotherapy with PIs resulted in the finding of significantly higher PD‐1‐positive CD4 central memory T cells, while IMiDs‐based chemotherapy just prior to lymphocyte apheresis prompted the finding of significantly lower PD‐1‐positive CD8 central memory, effector memory, and effector T cells in APs (Figure S4).

### 3.5 Univariate Analysis for Progression‐Free Survival

In order to assess the prognostic impact of CCR7‐positive CD8 T cells in APs, univariate analysis of PFS was performed (Table 3). The CCR7‐positive CD8 group had significantly better PFS compared to the low CCR7‐positive CD8 group (hazard ratio [HR] 0.23, 95%CI, 0.063–0.845, *p* = 0.028). Age at diagnosis, presence of EMD, presence of high‐risk cytogenic abnormality, poor PS at LDC, the status of penta‐refractory disease, or the presence of a treatment‐free interval over 3 months had no significant impact on outcome, while alkylator‐containing chemotherapy just prior to apheresis and treatment history of prior bispecific antibody over 3 months were associated with significantly worse outcomes (HR 8.49, 95%CI 2.057–35.06, *p* = 0.003; HR 6.84, 95%CI 1.885–24.78, *p* = 0.003, respectively).

**TABLE 3:** Univariate analysis for progression‐free survival.

	Univariate analysis		
	HR	95% CI	*p*‐Value
Age at diagnosis	1.05	0.984–1.120	0.144
Gender with male	1.35	0.452–4.027	0.592
Presence of extramedullary disease	1.12	0.386–3.258	0.833
Presence of poor‐risk cytogenetic abnormality	3.81	0.848–17.09	0.081
ECOG‐PS≧2 at LDC	2.87	0.957–8.621	0.060
Elevated ferritin level at LDC	3.27	0.693–15.43	0.134
The state of penta‐refractory	1.20	0.399–3.632	0.743
PIs‐based chemotherapy just before apheresis	1.28	0.426–3.870	0.658
IMiDs‐based chemotherapy just before apheresis	0.38	0.116–1.216	0.102
Alkylator‐containing chemotherapy just before apheresis	8.49	2.057–35.06	0.003
History of prior bispecific antibody use over 3 months	6.84	1.885–24.78	0.003
Presence of a treatment‐free interval over 3 months	0.35	0.108–1.125	0.078
Having high CCR7‐positive CD8 T cells	0.23	0.063–0.855	0.028

Abbreviations:ECOG‐PS, Eastern Cooperative Oncology Group performance status; IMiDs, immunomodulatory drugs; LDC, lymphodepleting chemotherapy; PIs, proteasome inhibitors.

## 4 Discussion

To our knowledge, this is the first report demonstrating that the patients with higher levels of CCR7‐positive CD8 T cells experienced significantly better PFS after the ide‐cel therapy. We also found that IMiDs‐based chemotherapy before lymphocyte apheresis was associated with a higher frequency of CCR7‐positive CD8 T cells with less PD‐1 positivity. From another report, maintenance therapy by lenalidomide (Len) after high‐dose therapy followed by autologous transplantation tends to increase the proportion of CD8 memory or central memory stem cells, however no alteration was observed in CD4 memory cells during the Len maintenance therapy [20]. Taken together, this information suggests that IMiDs‐based chemotherapy may relate to sustained relapse‐free survival through increasing CCR7‐positive CD8 T cell levels.

From our results, using IMiDs‐based chemotherapy just prior to apheresis is related to reduced PD‐1‐positive CD8 T cell levels in APs, while using PIs just before apheresis is related to higher levels of PD‐1‐positive CD4 central memory T cells in APs. From other reports, the PD‐1 positivity of CD8 T cells was stable during Len treatment [21], and also PD‐1 expression on both NK cells and cytotoxic T cells was reduced by prior use of Len treatment [22]. Prior use of PIs such as bortezomib promoted the activation of NF‐κB signaling on each subtype of T cells, leading to the transcriptional upregulation of PD‐1 in CD4 central memory T cells [23]. These results may suggest that the degree of T cell exhaustion in patients with RRMM varies according to their previous treatment history, reflecting the complex relationship between treatment history and the immunological microenvironment in each patient.

In a retrospective study including 171 (64%) patients treated with ide‐cel infusion, Gagelmann et al. proposed a prediction model of post‐BCMA targeting CAR‐T cell therapy (MyCARe model) [8]. They identified disease‐specific factors (presence of EMD or PCL, high‐risk cytogenetics), treatment‐specific factors (Len‐refractoriness), and inflammation‐specific factors (baseline ferritin at time of lymphodepletion) as independent predictors of relapse in 5 months after CAR‐T cell infusion, and they stratified the risk into three categories: 0–1 (low), 2–3 (intermediate), and 4 (high) [8]. Although we tried to apply the MyCARe model to our cohort, we were unable to find significant differences in each risk arm possibly due to the small number of cases and the more common occurrence of Len refractoriness in our cohort (21 patients [88%]), so we believe that a new stratification model based on T cell subtypes (or fitness) in apheresis samples may contribute to predict the therapeutic efficacy of CAR‐T cells. To clarify the predictive role of T cell subtypes in the ide‐cel therapy, we need further validation through a larger prospective study.

This study has several limitations. First, the small‐sample size of our cohort makes it difficult to exclude the effect of confounder or group imbalance. Moreover, this retrospective analysis may have affected interpretation of the survival results by limiting follow‐up duration. We plan to analyze more samples and have longer follow‐up to confirm the consistency of these results. Especially, our patients included relatively more heavily treated patients or patients with lower PS and more patients with high‐risk cytogenetic abnormalities than in previous reports [8, 24], presumably resulting in shorter overall PFS in our cohort. Second, we assessed PD‐1 positivity in T cells as a marker of exhaustion; PD‐1 is also a marker of activation [25]. However, T cell exhaustion is now understood to be a gradual process of activation that is modulated by metabolic and epigenetic processes [26]. So, this PD‐1 phenotype does not fully represent exhausted T cells. As another limitation, although the difference in T cell subsets is affected by not only the treatment history and but also tumor‐ or patient‐specific factors, we did not compare the change of T cell subtypes during each line of therapy and just before lymphocyte apheresis. Including temporal change of T cell subtypes in future analyses is warranted.

In conclusion, our results may aid the selection of specific patient populations that are likely to experience a more favorable outcome after the CAR‐T therapy and thereby contribute to the establishment of an optimal treatment sequence for immunotherapy and strategy for the CAR‐T cell therapy of RRMM.

## Author Contributions

Y.M., M.R., and S.I. conceived and designed the study. Y.M. and M.R. designed experiments and interpreted results. Y.M. generated data, performed data analysis, wrote the manuscript. M.R., T.E., T.N., Y.O., T.N., K.S, T.S., T.N., K.H, and T.S. provided advice and discussion.

## Conflicts of Interest

Shinsuke Iida has received honoraria and research funding from BMS. The other authors declare no conflicts of interest.

## Ethics Statement

This study was approved by the institutional ethics committee of Nagoya City University (protocol #70‐24‐0005).

## Supporting information



Supporting information

Supporting information

Supporting information

## Data Availability

The data that support the findings of this study are available on request from the corresponding author. The data are not publicly available due to privacy or ethical restrictions.
