# Supplementary material for: Prevalence of CCR7‐Positive CD8 T Cells as a Prognostic Factor in B‐Cell Maturation Antigen ‐Targeted Chimeric Antigen Receptor T Cell Therapy
Source: EJHaem. 2025 May 5;6(3):e70040. doi: 10.1002/jha2.70040 (PMC12051022; doi:10.1002/jha2.70040)
Supplement: Supplementary file 1 — Supporting information [file JHA2-6-e70040-s003.pptx]

## Slide 1
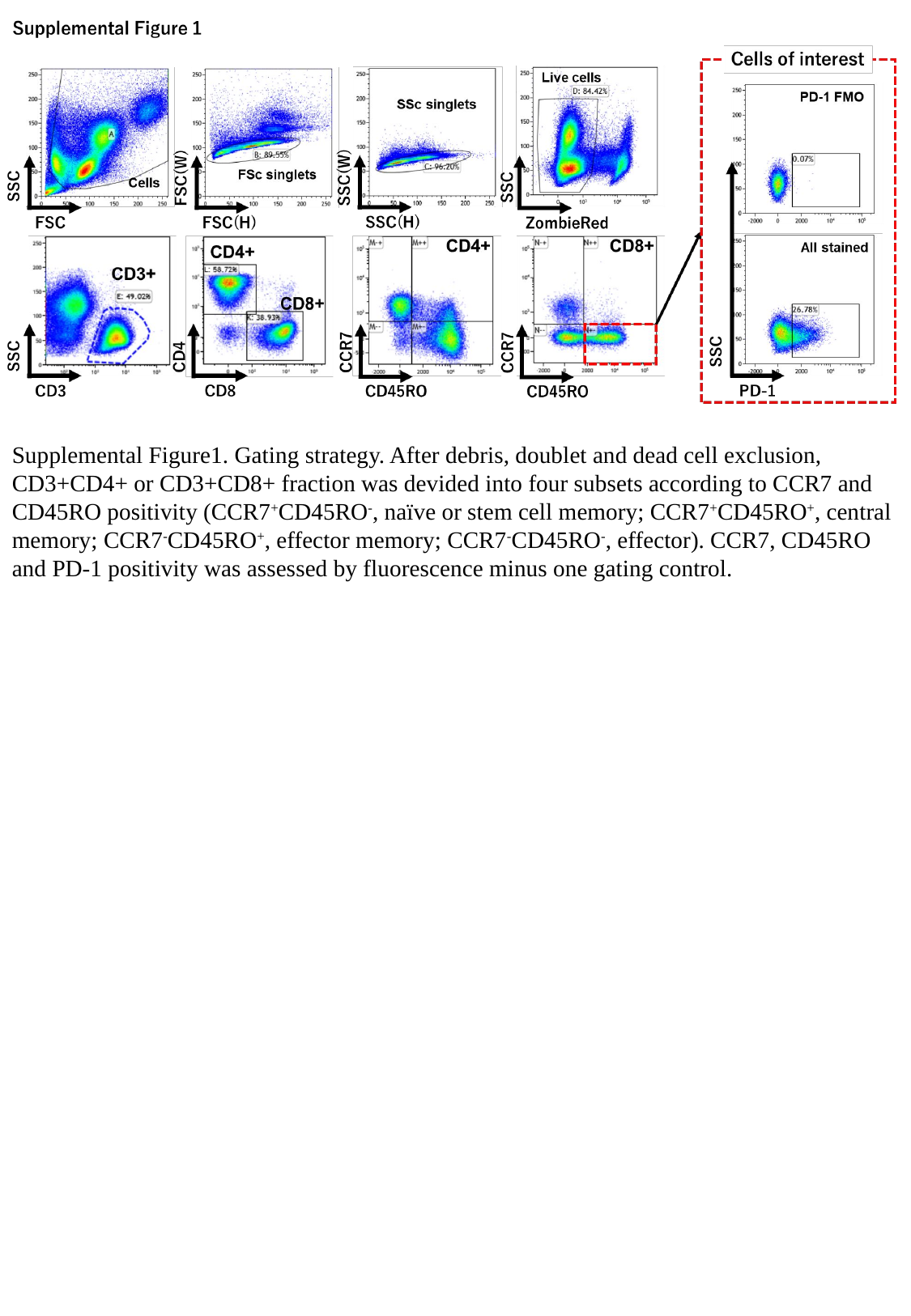

Supplemental Figure1. Gating strategy. After debris, doublet and dead cell exclusion, CD3+CD4+ or CD3+CD8+ fraction was devided into four subsets according to CCR7 and CD45RO positivity (CCR7+CD45RO-, naïve or stem cell memory; CCR7+CD45RO+, central memory; CCR7-CD45RO+, effector memory; CCR7-CD45RO-, effector). CCR7, CD45RO and PD-1 positivity was assessed by fluorescence minus one gating control.

## Slide 2
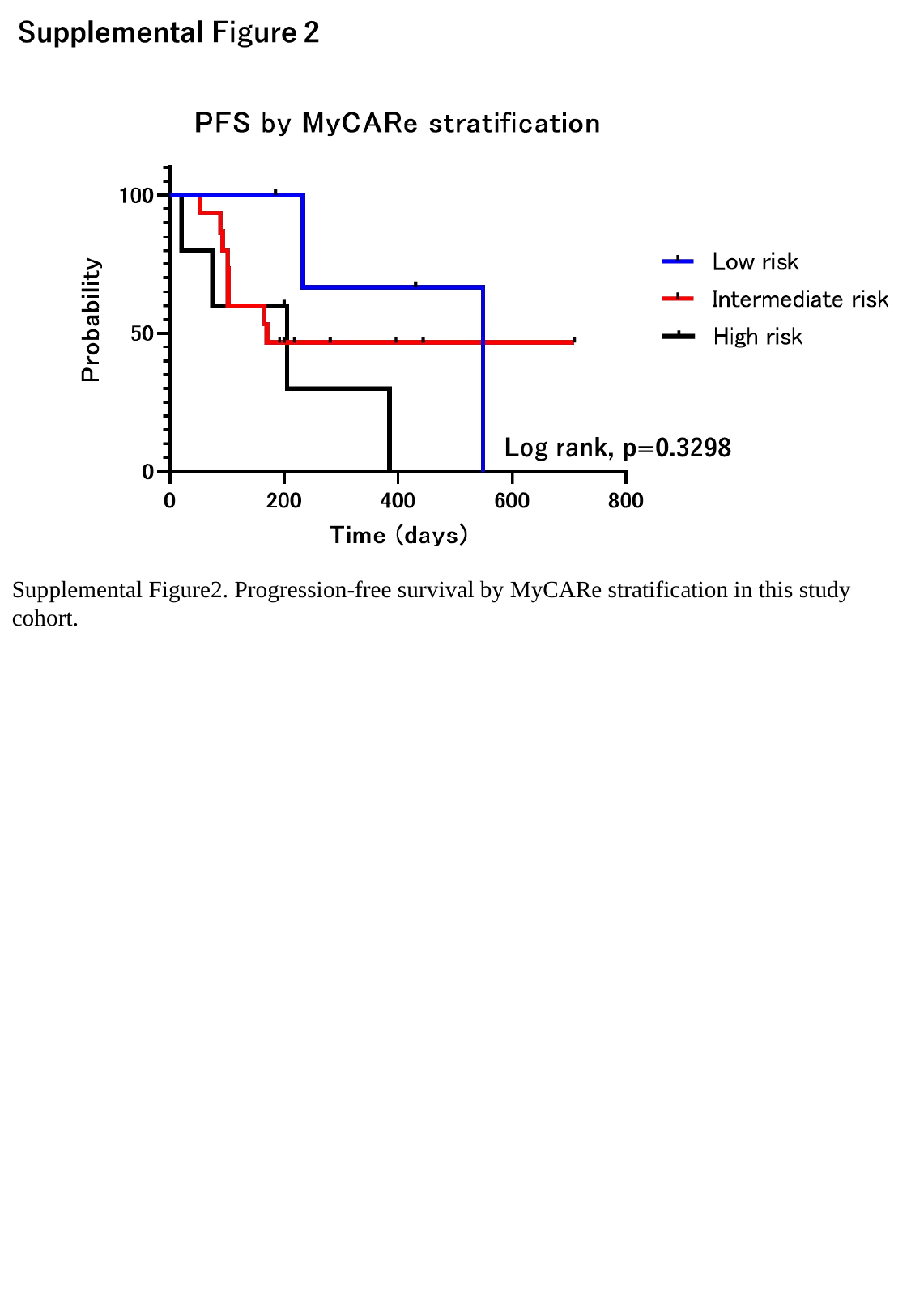

Supplemental Figure2. Progression-free survival by MyCARe stratification in this study cohort.

## Slide 3
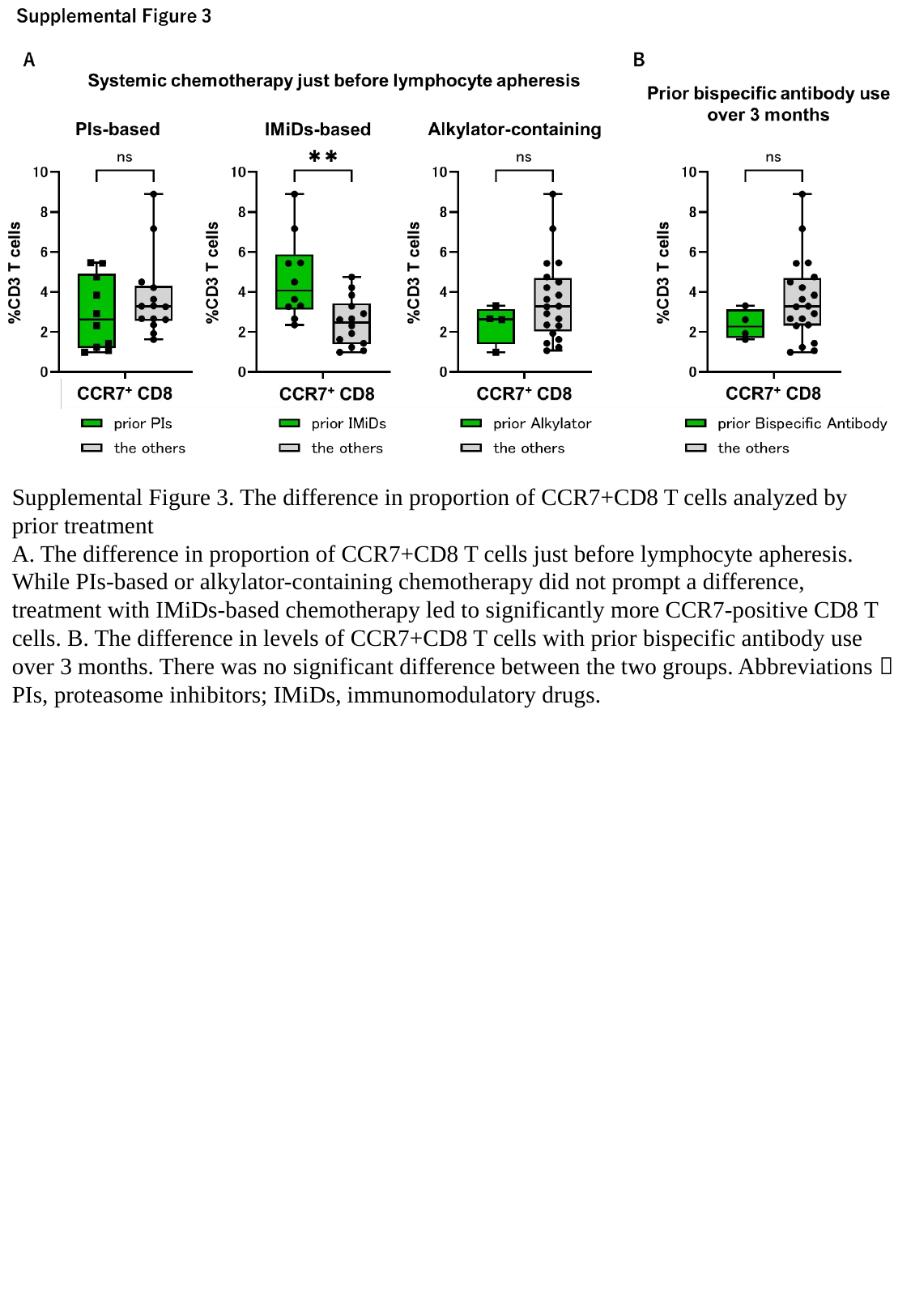

Supplemental Figure 3. The difference in proportion of CCR7+CD8 T cells analyzed by prior treatment
A. The difference in proportion of CCR7+CD8 T cells just before lymphocyte apheresis. While PIs-based or alkylator-containing chemotherapy did not prompt a difference, treatment with IMiDs-based chemotherapy led to significantly more CCR7-positive CD8 T cells. B. The difference in levels of CCR7+CD8 T cells with prior bispecific antibody use over 3 months. There was no significant difference between the two groups. Abbreviations：PIs, proteasome inhibitors; IMiDs, immunomodulatory drugs.

## Slide 4
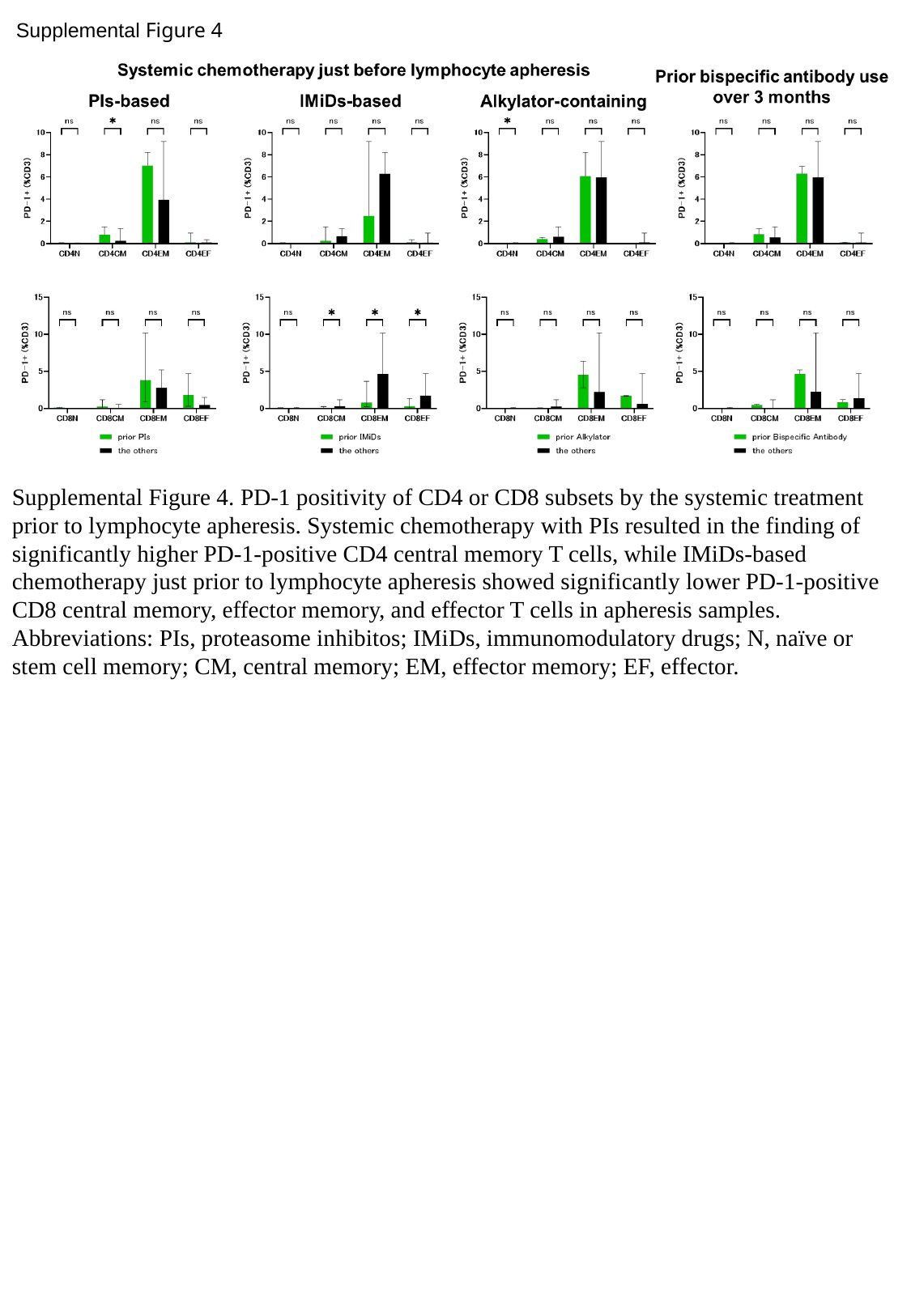

Supplemental Figure 4
Supplemental Figure 4. PD-1 positivity of CD4 or CD8 subsets by the systemic treatment prior to lymphocyte apheresis. Systemic chemotherapy with PIs resulted in the finding of significantly higher PD-1-positive CD4 central memory T cells, while IMiDs-based chemotherapy just prior to lymphocyte apheresis showed significantly lower PD-1-positive CD8 central memory, effector memory, and effector T cells in apheresis samples. Abbreviations: PIs, proteasome inhibitos; IMiDs, immunomodulatory drugs; N, naïve or stem cell memory; CM, central memory; EM, effector memory; EF, effector.
