# Supplementary material for: Prevalence of CCR7‐Positive CD8 T Cells as a Prognostic Factor in B‐Cell Maturation Antigen ‐Targeted Chimeric Antigen Receptor T Cell Therapy
Source: EJHaem. 2025 May 5;6(3):e70040. doi: 10.1002/jha2.70040 (PMC12051022; doi:10.1002/jha2.70040)
Supplement: Supplementary file 2 — Supporting information [file JHA2-6-e70040-s002.docx]

**Supplemental Table 1.** Treatment response of all study participants and long or short responder groups.

|  | **All participants**  **(n=24)** | **Long responders**  **（n=14）** | **Short responders**  **（n=10）** | **p value** |
| --- | --- | --- | --- | --- |
| Best overall response |  |  |  | 0.002 |
| sCR | 7 (29.2%) | 7 (50.0%) | 0 (0.0%) |  |
| CR | 5 (20.8%) | 4 (28.6%) | 1 (10.0%) |  |
| VGPR | 6 (25.0%) | 3 (21.4%) | 3 (30.0%) |  |
| PR | 3 (12.5%) | 0 (0.0%) | 3 (30.0%) |  |
| SD | 3 (12.5%) | 0 (0.0%) | 3 (30.0%) |  |
| PD | 0 (0.0%) | 0 (0.0%) | 0 (0.0%) |  |
| CR rate^¶^ | 12 (50.0%) | 11 (78.6%) | 1 (10.0%) | 0.003 |
| Overall response rate^§^ | 21 (87.5%) | 14 (100.0%) | 7 (70.0%) | 0.059 |

Abbreviations：sCR, stringent complete response; CR, complete response; VGPR, very good partial response; PR, partial response; SD, stable disease; PD, progressive disease.

^¶^CR rate was defined as a complete response or a stringent complete response.

^§^Overall response rate was defined as a partial response or better.
