# Supplementary material for: Prevalence of CCR7‐Positive CD8 T Cells as a Prognostic Factor in B‐Cell Maturation Antigen ‐Targeted Chimeric Antigen Receptor T Cell Therapy
Source: EJHaem. 2025 May 5;6(3):e70040. doi: 10.1002/jha2.70040 (PMC12051022; doi:10.1002/jha2.70040)
Supplement: Supplementary file 3 — Supporting information [file JHA2-6-e70040-s001.docx]

**Supplemental Table 2.** Treatment response by the frequency of CCR7-positive CD8 T cells.

|  | **High CCR7^+^CD8**  **（n=9）** | **Low CCR7^+^CD8**  **（n=15）** | **p value** |
| --- | --- | --- | --- |
| Best overall response |  |  | 0.196 |
| sCR | 5 (55.6%) | 2 (13.3%) |  |
| CR | 2 (22.2%) | 3 (20.0%) |  |
| VGPR | 2 (22.2%) | 4 (26.7%) |  |
| PR | 0 (0.0%) | 3 (20.0%) |  |
| SD | 0 (0.0%) | 3 (20.0%) |  |
| PD | 0 (0.0%) | 0 (0.0%) |  |
| CR rate | 7 (77.8%) | 5 (33.3%) | 0.089 |
| Overall response rate | 9 (100.0%) | 12 (80.0%) | 0.266 |

Abbreviations：sCR, stringent complete response; CR, complete response; VGPR, very good partial response; PR, partial response; SD, stable disease; PD, progressive disease.

^¶^CR rate was defined as a complete response or a stringent complete response.

^§^Overall response rate was defined as a partial response or better.
